# Assessing and Addressing US Health Security Risks

**DOI:** 10.1089/hs.2016.0102

**Published:** 2017-02-01

**Authors:** Crystal R. Watson

There are a range of worrisome threats to US health and national security—worrisome because they have the potential to cause widespread disruption and damage to the public's health and to the US and global economies. These threats include major natural hazards like large earthquakes and hurricanes and infectious disease pandemics; accidental threats, including technological failures, such as nuclear power plant disasters like Three Mile Island and Fukushima; and intentional attacks by thinking adversaries, including terrorist use of biological, chemical, or nuclear/radiological weapons.

Securing the United States against threats to health and security inherently involves tradeoffs. Not every health security–related threat carries the same level of risk to the country, and not every government program addressing those threats can have top priority.

So, with many and varied health security threats and with limited resources to address them, it is important for the United States to have a clear process for determining risks and tradeoffs in a way that affords the public the most protection we can offer. Utilizing our best risk assessment methodologies is an important part of determining risk and setting national priorities.

Risk assessment is useful both for understanding the range of possible health security challenges the country might face (eg, possible disaster/outbreak scenarios) and for gathering data and knowledge about threats, which may help security and public health experts prepare and respond if something does occur. It involves a systematic evaluation of the likelihood (probability) of an event occurring, as well as of the consequences that result when a disaster does happen (eg, illnesses, injuries, deaths, and economic disruption).

A purposeful risk assessment process can help leaders compare the severity of different types of health security risk and can inform decisions about where funding and resources are most needed. Risk assessment allows decision makers to view a wider range of risk scenarios and examine them systematically. Without a formal process to assess risk, decision makers often rely on personal experience, which is inherently limited (we are only human after all). Conducting a risk assessment that systematically compares likelihood, vulnerability, and consequences for different scenarios helps reduce individual and group biases, which can prevent decision makers from seeing the full picture of risk before decisions are made.

Risk assessment is currently carried out by a variety of federal, state, and local government agencies for important but varied and delimited purposes. For example, the Department of Homeland Security (DHS) conducts chemical, biological, radiological/nuclear, and integrated CBRN terrorism risk assessments to understand the relative likelihood and consequences of terrorist use of any CBRN agent or weapon to attack the United States; the Department of Defense (DoD) assesses the variety of threats to military personnel and assets in the United States and abroad; and state and local public health and emergency management agencies undertake threat and hazard identification and risk assessments to understand and prepare for the most likely and consequential disasters at the local level.

All of these, and other ongoing risk assessment efforts, are important and contribute to our understanding of specific threats, vulnerabilities, and risks to national security and human health. However, they are conducted separately, with limited coordination among them. There is not currently a national-level risk assessment that compares all of the threats to national health security that we worry about, all in one place, so that they can be compared, contrasted, and the results used to help determine national health security priorities for our government.

Yet, while a national-level cross-hazard strategic risk analysis does not currently exist for the United States, there are past efforts here and current practices in other countries to look toward for inspiration. For example, in 2011, DHS conducted a one-time Strategic National Risk Assessment (SNRA) in support of Presidential Policy Directive 8 (PPD-8), to inform creation of a National Preparedness Goal, System, and Report. Through this process, the SNRA identified and gathered likelihood and consequence data on “national-level events,” including naturally occurring hazards, accidents and technological disasters, and intentional events, and conducted a subject matter expert–driven risk assessment to determine their relative risk. This SNRA process was conducted at the national level, but it was not tied directly to program planning and has not been revised or repeated since that time.^1^

Outside of the United States, both the Canadian and British national governments produce annual risk assessments of “civil emergencies” or “all-hazards” that include natural, accidental, and malicious/intentional disasters.^2,3^ These assessments incorporate historical data, threat information, and expert knowledge and opinion and provide rankings and recommendations for action to address risks identified in the process. The results of these assessments are intended to inform policy formulation and resource decisions that drive priority setting for national agencies responsible for executing programs.

A national risk assessment, such as the examples described above, could be a powerful tool to aid in decision making, but it will also have its limitations. Risk analysis depends on data and evidence, which are not always available (especially for rare events that we seldom or have not yet experienced as a country). As a result, conclusions from risk assessment can be highly uncertain and should not be the sole basis of any decision. However, having a national risk assessment would make it more likely that we will identify and then allocate resources effectively to prepare for and respond to the next health security threats we face.

## Recommendation

➢ **Establish a national risk assessment process for the United States.**

What is needed is a comprehensive, strategic-level US risk assessment that identifies and compares all major threats to the homeland, including all types of terrorism, natural disasters, naturally occurring epidemics, and technological disasters that would have a significant impact on our national security if they were to occur. The White House has a unique government-wide perspective on health security risk and a unique position from which to coordinate and direct federal health security programs. Therefore, it is appropriate for the White House to initiate and lead a national risk assessment process. A first step in this process would be for the White House to convene an advisory committee, including experts in risk assessment, public health, medicine, and national security, to determine the agency or agencies that should implement the risk assessment, what risk analysis methods will be used, and how often the assessment should be updated and revised.

There are a number of risk assessment methodologies that could be applied to a national risk assessment, from a fully quantitative analysis to a fully qualitative assessment based solely on expert judgment. The examples above from the US, UK, and Canada used various combinations of quantitative and qualitative methods, combining data or data-driven estimates collected about likelihood/frequency and consequences of events with subject matter judgment about how those identified risks should be ranked and prioritized. A similar method would likely work well for a new US national risk assessment.

Finally, once this risk assessment process is set and is being used, it will be important to revise the risk assessment, and review the methods, inputs, and focus of the assessment regularly to ensure that it is rigorous and helpful to understanding and addressing health security risk.
